# Spotlight on Calcipotriol/Betamethasone Fixed-Dose Combination in Topical Formulations: Is There Still Room for Innovation?

**DOI:** 10.3390/pharmaceutics14102085

**Published:** 2022-09-29

**Authors:** Francesca Selmin, Silvia Franzè, Antonella Casiraghi, Francesco Cilurzo

**Affiliations:** Department of Pharmaceutical Sciences, Università degli Studi di Milano, Via Giuseppe Colombo, 71, 20133 Milan, Italy

**Keywords:** chemical compatibility, epidermal barrier, microneedles, nanovectors, polyaphron dispersion, psoriasis model, rheological properties, skin permeability, transepidermal water loss, topical formulation

## Abstract

Psoriasis is a lifelong disease which requires treatment adherence for successful management. Considering the complexity of this pathology, the combination of active pharmaceutical ingredients with a synergistic mechanism of action can improve the safety and efficacy of the treatment with respect to the conventional monotherapy. Moreover, a fixed dose of therapeutic agents in a topical formulation offers the possibility to simplify administration, reduce the doses of each active ingredient, and improve patient’s compliance. Among the first-line treatments in mild to moderate psoriasis, the formulation of calcipotriol (Cal) and betamethasone dipropionate (BD) in a single vehicle is challenging due to their chemical incompatibility in an aqueous environment and the formation of degradation products. Based on these considerations, this review aims to provide an overview on the biopharmaceutical properties of Cal/BD fixed-dose combination products available on the market (namely ointment, oleogel, foam, and O/W cream), highlighting also the novel approaches under evaluation. The main differences among topical formulations are discussed considering the different features of the anatomic districts involved in psoriasis and the patient’s adherence. Moreover, since in vitro experiments are fundamental to evaluate the skin permeation profile during the development of an efficacious medicinal product, special emphasis is given to models proposed to mimic psoriatic lesions.

## 1. Introduction

Psoriasis is a common inflammatory skin disorder affecting approximately 125 million people around the world. This disease is characterized by hyperproliferation and aberrant differentiation of keratinocytes with a complex immunogenetic basis. The clinical manifestations, namely redness and skin lesions, are often seen at the extensor surfaces of the sacral areas, elbow, knees, and scalp. Although there is no cure for psoriasis, there are multiple effective treatment options. Potent corticosteroids with anti-inflammatory properties are considered the cornerstone of topical treatment for patients with mild to moderate psoriasis. However, it is required to formulate topical corticosteroids in a vehicle with a suitable potency and ease to spread in different body sites in order to avoid potential side effects. As an example, betamethasone as such has limited success when applied topically since only 12–14% of the administered dose is absorbed. Therefore, esters [1], such as betamethasone-17,21-dipropionate (BD), or betamethasone-17-valerate (BV), must be used because the increased lipophilicity can enhance the local bioavailability. Vitamin D analogues, and in particular calcipotriol (Cal), have also been proposed to treat psoriasis since Cal inhibits keratinocyte proliferation, promotes their differentiation through epidermal layers, and suppresses proinflammatory T-cell cytokine production [2]. However, the efficacy as monotherapy is limited and treatment discontinuation due to skin irritation often occurs. The combination of BD and Cal improves the clinical outcomes for the patients because it has a synergistic effect: Cal mitigates atrophy and decreased epidermal barrier integrity caused by BD and BD counteracts the irritant effects of Cal. The first treatments of psoriasis with a combination of Cal and BD were initially carried out by applying them separately twice a day or sequentially. However, Cal has a limited penetration ability through the stratum corneum into the epidermis, requiring proper strategies of enhancement of skin penetration [3]. Fixed-dose combination products, namely formulations combining Cal and BD in a single vehicle, have been developed to increase the effectiveness of drugs, simplify administration, and improve patients’ compliance.

From a pharmaceutical perspective, the development of a fixed-dose combination product requires a careful evaluation of unintended interactions between active substances and/or excipients leading to a lack of activity and/or adverse effects. In the case of betamethasone esters, they are susceptible to an acid- and base-catalyzed isomerization and the isomer presents a lower potency. As an example, in BV, the acyl group migrates from position C-17 to the more stable C-21 position [4] with the concomitant potency reduction of about 1/15th [5]. Furthermore, the dilution with other vehicles (i.e., hydrophilic base creams or other topical medicinal products) can cause the chemical alteration of betamethasone esters [1], modifying their biopharmaceutical performances. Cal is sensitive to oxidizing agents and acidic residues; it reacts with alcohols and easily undergoes epimerization processes. When water is present, Cal requires a pH value above 8 for maximum stability; and again, the mixing with other drugs or topical products leads to a decrease in pH (i.e., salicylic acid or corticosteroids), causing a dramatic reduction in Cal content [6]. Based on these stability data, the formulation of Cal/BD fixed-combination products requires different pH values in aqueous environments to avoid the formation of degradation byproducts and, therefore, limit the possible choice among topical formulations. Moreover, the bioavailability should be similar to that of monotherapy products, keeping in mind the possible local side effects of a corticosteroid [7]. Finally, the formulation should be chosen also considering the patient’s preference even among the youngest: the formulation should be well-tolerated with an acceptable safety profile also for children and adolescents. In this patient population, adherence is even more important since psoriatic adolescents deal with a potentially disfiguring and chronic disease that could permanently impair their psychological development [8,9].

In the light of these considerations, the current narrative review deals with the challenges of designing Cal/BD fixed-dose combination topical formulations in the attempt to relate their main features to patient’s preferences and, therefore, to the impact on clinical outcomes in daily practice. First, the attention is focused on the medicinal products available on the market (i.e., ointment, oleogel, suspension, foam and, the newest one, cream). The second part of this review covers the academic and industrial efforts to satisfy the general request for new topical formulations with improved efficacy, cosmetic acceptance, and easiness to be applied on different body parts [10].

## 2. Skin Barrier Disruption in Psoriasis

The ability of a given active ingredient to penetrate into the skin depends on several factors, among which the drug physico-chemical properties (i.e., molecular weight, logP, melting point), thermodynamic activity into the vehicle, and the skin barrier properties dictate the efficacy of new topical therapies. Dysregulation due to radiation, infection, and inflammatory processes can affect the skin barrier function and contribute to many inflammatory skin disorders. In the case of psoriasis, uninhibited proliferation and abnormal differentiation of keratinocytes induce the formation of plaques, the main features of which are hyperkeratosis (i.e., the thickening of the stratum corneum) and acanthosis (i.e., the thickening of other epidermal layers). In plaques, the cell density and differentiation are two to five times and five to seven times faster than normal skin, respectively [11]. Moreover, the disruption of epidermal tight, gap, and adherens junction proteins are meant to stem in the skin barrier dysfunction [11]. This morphological variation of skin organization contributes to the concomitant increase in the transepidermal water loss (TEWL) and reduction in the water binding capacity, which are responsible of the decrease in stratum corneum hydration, typical of the psoriatic lesions [12]. 

Barrier disturbance derives also from the dysregulation in the composition of the stratum corneum extra-cellular matrix and, in particular, the ceramide subtypes. Psoriatic skin presents a minimal reduction in acylceramide levels and diminished long periodicity phase, a unique lamellar arrangement of skin lipids [13,14]. Again, these variations are correlated with TEWL and have an impact on the drug permeability. As an example, the permeability of theophylline and indomethacin through model membranes reproducing the altered lipid nanostructures (i.e., lack of periodicity lamellar phase, low ordered lipids, and low orthorhombic chain packing) was increased with respect to that obtained using membranes with a composition closest to that of the native skin lipid barrier [15]. Skin lesions have also a reduced level of short chain fatty acids and an increased content in cholesterol in comparison to healthy skin. Hence, the penetration and permeation of a substance into psoriatic and normal skin can differ both in the extent and rate due to the differences in the overall lipid content and thickness of epidermal layers. One would expect that the penetration-barrier deterioration may accelerate drug delivery into the skin, augmenting the risk of over-absorption. This was confirmed by Oestmann et al. as they demonstrated a variation in TEWL in psoriatic skin and a consequent reduction in barrier activity correlated to clinical signs of scaling [16]. 

Due to these massive differences between healthy and psoriasis skin, the in vitro or ex vivo evaluation of drug permeability during the design of a topical formulation is not exempt of challenges since the availability of models able to mimic the reduced barrier function is very limited. One of the in vivo models proposed to this purpose is based on the topical use of imiquimod which promotes a dermatitis recalling several psoriasis-like signs at epidermal, vascular, and immune levels. This model was used to evaluate the percutaneous absorption of four drugs frequently used to treat psoriasis, with different physico-chemical characteristics, namely Cal, 5-aminolevulinic acid, tacrolimus, and retinoic acid. After imiquimod treatment the skin absorption was increased for each drug by different folds, and it was concluded that the lipophilicity was the main parameter in the dominated drug-permeation enhancement in the psoriatic lesions [17].

On the other hand, the thicker the epidermis layer, the longer the pathway through which a drug has to diffuse. Recently, imiquimod-induced psoriasiform dermatitis in wild-type mice was used to evaluate the impact of changes in some skin parameters on caffeine permeability. An increased skin thickness and the impairment of epidermal barrier function were measured 48 h after imiquimod topical application; afterwards, the psoriasiform dermatitis was induced until 96 h. The caffeine permeability significantly increased compared to the petrolatum treatment, used as control, at the first stage of the psoriatic process, but at the more advanced stage (96 h), a reduced permeability appeared [18].

Despite these data, modeling psoriasis is still a challenge because this pathologic condition does not occur in laboratory animals and other models are poorly described. An attempt to bridge this gap, new reconstructed human skin equivalents (RSE) model proposed by Mok, B.R. et al. Adding IL-17A into the culture medium during RSE preparation, they succeeded in the development of a tissue that exhibites psoriatic epidermal characteristics. This model can be potentially used in investigating the pathogenesis of inflammatory skin diseases or testing drugs and formulations to treat this class of disease [19]. However, due to the paucity of data, excised healthy skin, both from human abdomen or pig ear, is used to test and compare formulations. 

## 3. Selection of Vehicles in the Design of Fixed-Dose Combination Products

### 3.1. Topical Vehicles Available on the Market

As mentioned, the choice of a formulation suitable for Cal/BD fixed-dose combination is limited to their chemical incompatibility and limited solubility in the aqueous environment. Hence, vehicle components should be non-aqueous, compatible with both drugs, and able to deliver them into the skin at a comparable rate. Nowadays, medicinal products approved for the treatment of psoriasis are available in five topical semisolid formulations: ointment, oleogel, suspension, foam and, the newest one, cream. 

Cal/BD ointment was the first formulation approved in the EU and USA to treat plaque psoriasis. This formulation is very effective in glabrous areas—i.e., soles and palms—or on skin with short or sparse hair, as it is difficult to wash off; moreover, it is reported that ointments can ameliorate nail matrix signs in nail psoriasis, which is estimated to have a lifetime incidence of 80–90% among patients and a great impact on the quality of life since these lesions involve one of the most visible areas of the body [20,21]. 

Generally speaking, the pharmacopoeia defines ointment as a monophasic system made of lipophilic excipients, such as petrolatum and mineral oil, or petrolatum and waxy/fatty alcohol combinations. Due to the lipophilic nature, ointments can induce skin occlusion, improving the penetration of the active components and ameliorating the dry state of the skin; moreover, the ratio and grades of lipophilic excipients provide the desired viscosity and/or spreadability of the finished product. At the same time, ointments can be perceived as unpleasant and difficult to apply, affecting the patient’s adherence to the treatment. In the design of this dosage form, Simonsen et al. evaluated the ability of several excipients to optimize the solubility of Cal and BD and their skin permeability [22]. Among the possible excipients, propylene glycol was discarded since it may contain some acidic impurities enabling the catalyzation of Cal epimers and the formation of impurities, such as calcipotriol propylene glycol ethers. Furthermore, the addition of a buffer system at pH above 8, which is the easiest strategy to avoid this problem, should be avoided because BD would be easily hydrolyzed into betamethason. The formulations containing lanolin or isopropyl myristate were also discarded because no or negative effects on the in vitro permeation rate were observed. Only polyoxypropylene-15 stearyl ether (PSE) was considered worthy of further investigation since at 5% concentration it allowed researchers to obtain a permeation rate comparable to those of marketed products [22]. 

As the treatment of psoriasis of the scalp is a challenge due to the thickness and the limited accessibility of the lesions, lipophilic gels or suspensions can be conveniently applied [23]. In the early development process of the oleogel, Cal penetration through the skin was optimized using the psoriasis plaque test [24] which allows simultaneous topical application of different drugs, or formulations on multiple test sites in the same psoriasis patient. Hence, recruiting a limited number of subjects, it is possible to directly compare the anti-psoriatic effect of the proposed treatment [24]. The bio-assay data evidenced that changing the oleogel base from isohexadecane to mineral oil increased the bioavailability of both drugs. However, the final formulation containing mineral oil and 16% PPG-15 stearyl ether was preferred [25].

A revolution in the topical treatment of psoriasis has been established in 2015 after the approval of an alcohol-free foam. This dosage form undergoes a drastic metamorphosis/transformation upon application on the stratum corneum. Indeed, due to the presence of a propellant (i.e., dimethyl ether), a liquid can generate a foam: this modification of the formulation increases the drug thermodynamic activity, enhancing the skin penetration and, therefore, the bioavailability with respect to conventional dosage forms [26]. The supersaturated system was demonstrated to be stable at least 26 h after propellant evaporation since no crystals of BD or Cal were detected by Raman imaging [27]. Moreover, the large amount of surfactants, which can enhance the penetration, and the occlusive properties of the foam may improve the dermal bioavailability of both drugs [27].

**Figure 1:**
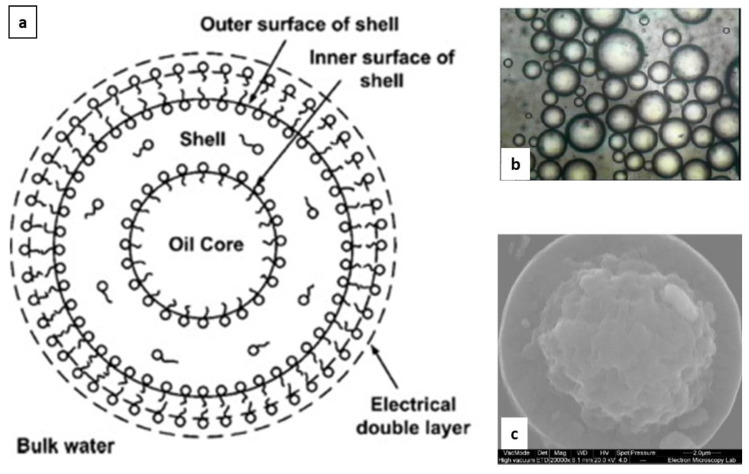
An aphron consists of a core of apolar solvent encapsulated in an outer shell with a multilayer structure of oil, water, or water-soluble surfactant which stabilizes the core in the continuous phase (panel (**a**)). The multimolecular layer forms a robust shell around oil droplets, improving the colloidal stability of aphrons (panel (**b**), adapted from [29]). The size of the polyaphron droplet is comprised in the micron range. The cryo-SE microphotograph of a fractured aphron shows also the presence of a central, rough region surrounded by a smooth outer shell (panel (**c**), adapted from [30]).

In 2020, a O/W cream was approved to treat plaque and scalp psoriasis in adults. To overcome chemical incompatibility, instability in aqueous environment and solubility issues of BD and Cal, the polyaphron dispersion technology (PAD^TM^ technology) was exploited. In detail, aphron (also referred to as gel emulsions, foam-like emulsions, bi-liquid foams, high internal phase emulsions (HIPEs), or high internal phase ratio emulsions (HIPRE)) were postulated to consist of a micron-sized oily droplet forming a core encapsulated in a multi-layer structure of water, surfactants, and oil (Figure 1). The multilayer structure improved the colloidal stability of aphrons and limited the partitioning of substance(s) from the core to the continuous aqueous phase [28]. 

Nowadays, the multi-layer organization is described as a dynamic nanoscale 3D bicontinuous structure involving not only the surfactant, but also bond water and oil [31]. 

In a Cal/BD fixed-dose combination product, the cream comprises two sets of aphrons, in which either BD or Cal are separately encapsulated, in an aqueous continuous phase consisting of a carbomer hydrogel at pH around 7.75. According to the compositions described in the patent, the cream can contain from 70% to 90% of the dispersed oil phase and as little as 0.5% (reaching a maximum of 3%) of surfactant/polymer of the total weight [32,33]. Indeed, the distribution of surfactants in aphrons allows the stabilization of the inner oil core; and at the same time, the total amount required to avoid creaming is from several fold up to 30-fold lower than in conventional creams and lotions with a comparable content of oil. This peculiar feature allows avoiding the wash-off of epidermal lipids which leads to the disruption of the skin barrier properties and irritation [34]. Moreover, the lower the surfactant levels, the lower the solubility of lipophilic drugs, such as Cal and BD, in the aqueous phase; in other words, the overall chemical stability of active ingredients is improved over the storage period [31]. The cream composition is also responsible for the greater skin flux compared to the marketed Cal/BD oleogel and ointment (Table 1).

Regarding the flux reported in papers or estimated from the release profiles (Table 1), it should be noted that in all cases, in vitro skin permeation data are determined using the healthy skin of either humans or pigs, without considering the differences in thickness of the stratum corneum in the various districts of the human body. Indeed, the anatomic site is another factor that can influence the percutaneous absorption, as demonstrated by the amount of ^14^C-hydrocortisone excreted in the urine after application on different areas. For example, the absorption through the palm resulted in being 0.83 lower than that of the forearm, while the amount absorbed after application on the scalp was 3.5 higher [36]. 

Among the innovative drug delivery systems under clinical investigation, microneedle patches may be appealing to psoriatic patients to solve issues related to the plaque thickness. Microneedles are minimally invasive devices consisting of an array of fine polymeric needles which can result in micron-sized pores upon insertion into the skin (“poke and patch” approach). Pain cannot be felt due to the micron-sized structure and the skin recovers easily after removing microneedles. Thanks to this feature, they offer an opportunity to improve the bioavailability of various therapeutics and to administer future vaccines, particularly in the developing world. Most of microneedles available on the market are indicated for cosmetic use, but size, shape, height, and distribution of microneedles have been a matter of investigation, since these features influence the duration of the channels in the stratum corneum. For instance, biodegradable microneedles with different height were fabricated by hot-press method and inserted into mouse dorsal skin to evaluate their safety: the disappearance of marks caused by microneedles with a height of 650 μm and 300 μm occurred after 20 min and 5 h, respectively [37]. There are two main limitations in this approach to treat psoriasis. First, microneedle patches may only be applied to small, localized psoriatic plaques in contrast to extensive psoriatic lesions. Then, the mechanical resistance of the microneedle array should be carefully evaluated since the hyperkeratotic and acanthotic nature of plaques could limit microneedle penetration. 

To treat skin pathologies using the “poke and patch” approach, the topical vehicle can be applied directly on the skin and then the device can be inserted; or the skin can be pre-treated with microneedles before applying the formulation. Both protocols were tested to improve the permeation of Cal through a psoriasis-like skin inflammation model in ear and dorsal skin of mice; signs of skin inflammation, including skin and epidermal thickening, inflammatory cell infiltration, and spleen weight gain were also monitored. Even if both protocols provided satisfactory results, the pretreatment with microneedles was better [37]. In a recent pilot study [38], hyaluronic acid microneedle patches (Therapass^®^ RMD-6·5A; Raphas, Cheonan, Korea) were inserted over the thickest areas on one or more resistant psoriatic plaques at night after topical application of the commercial ointment and removed in the morning. After 1 week of treatment, the complete resolution of lesions was evident in two patients (20%) and eight patients (80%) were very satisfied or satisfied. No side effects, e.g., irritation, infection, or aggravation of pre-existing psoriasis, were noted [39]. Despite these positive outcomes, the small number of enrolled patients and lack of a placebo control group are major limitations of this clinical trial.

### 3.2. Topical Vehicles in R&D

To improve the therapeutic potential of topically applied drugs, nanotechnology-based drug delivery systems represent a promising strategy since they can deliver drugs directly in the epidermis, eventually targeting specific pathways involved in psoriasis disease. Different nanocarriers have been investigated to treat skin pathologies, including nanoemulsions, liposomes, and further generations of highly deformable liposomes s specifically designed for (trans)dermal application purposes, such as transfersomes and ethosomes, but also nanostructured lipid carriers (NLC) and solid lipid nanoparticles (SLN). To the best of our knowledge, fixed-combination Cal/BD was loaded only into solid-lipid nanoparticles (SLN). Precirol ATO 5 as a lipid and Pluronic F-68 as a surfactant resulted in the optimal composition to prepare SLN sizing around 200 nm with high loading of both drugs and good colloidal stability. The efficacy of SNL incorporated in a Carbopol gel was tested by mouse tail model. The results showed a decrease in the epidermal thickness and an increase in the melanocyte count associated with a high level of drugs in the epidermis and dermis in comparison to commercial ointment [40]. 

The paucity of work dealing with the feasibility of loading Cal/BD fixed-dose combination into a nanocarrier could be principally due to drug incompatibility and not to lack of efficacy of nanovectors since these drug delivery systems, and in particular deformable liposomes, have shown a high capability of penetration in the skin, allowing to maximize the drug dose at the target tissue. Indeed, nanocarriers loaded by a wide range of active ingredients have provided encouraging results in the treatment of psoriasis, demonstrating their efficacy and improved safety [41,42]. As an example, cyclosporine-loaded liposomes resulted in having superior efficacy compared to conventional formulations in treating psoriasis both in an imiquimod-induced plaque psoriasis model and in a single-centered randomized clinical trial carried out in 38 patients with chronic plaque psoriasis [43]. Sterically stabilized liposomes composed of distearoylphosphatidylcholine (DSPC) and sodium cholate were able to enhance the penetration of Cal in the skin, in a size-depended manner [44]. 

Other strategies were proposed to co-administer drugs to face potential incompatibility issues and, meanwhile, to control the drug-release rate and depth of skin penetration. For instance, it is well-known that the topical administration of methotrexate allows the reduction of the psoriasis score at a very low range. To ameliorate its biopharmaceutical properties (i.e., poor penetration and ionization at physiological pH), methotrexate loaded ethosomes were designed and dispersed in a gel matrix of salycilic acid. This expedient allowed a higher residence time and increased penetration of ethosomes through the skin with a higher retention percentage with respect to the methotrexate solution. The overall results of an animal study showed a significant improvement of skin conditions with very slight keratosis [45].

Clobetasol propionate (CP) is another potent alogenated glucocorticoid with an optimum chemical stability at pH 4–6 since at higher pH values the ester is hydrolyzed. As proof of concept, CP/Cal fixed-dose combination was loaded in a nanoemulsion-based gel which increased the drug availability in the viable layers of the skin with respect to control formulations, assuring a good healing process in psoriasis and reducing skin irritation [46]. In another work, cyclosporine A and Cal were co-encapsulated in highly hydrophobic carriers, namely SLN and NLC. In particular, NLC were able to entrap a higher amount of both hydrophobic drugs in the presence of liquid lipids in the composition and showed also a higher uptake by the dermal cells and deeper penetration into the viable layers of the skin when compared to the others and also marketed formulations. Finally, the application of the gels led to a decrease of inflammation [47].

## 4. Topical Vehicles and Patient’s Preferences

Despite the availability of innovative drug delivery systems for the topical treatment of psoriasis, poor treatment outcomes are determined by poor adherence, namely the process by which a patient takes his/her medicines as prescribed. Adherence is dictated by three steps: initiation, implementation, and discontinuation. The first refers to the moment at which the patient takes the first dose of a prescribed medicine; it is followed by the implementation of the dosing regimen; and discontinuation, when the next dose to be taken is omitted and no more doses are taken [48]. 

Patients with psoriasis consistently report that topical treatment is one of the most negative aspects of the disease [49] and, even if they should be strongly motivated to treat their disease, poor adherence is probably higher than 40% [50,51]. To ameliorate adherence, a model has even proposed to explain the complex interactions among the disease, patient, treatment characteristics, adherence, and outcomes [52]. Furthermore, validated questionnaires are useful to evaluate the patient’s acceptance of a topical therapy or to compare preferences among formulations [31]. 

**Figure 2:**
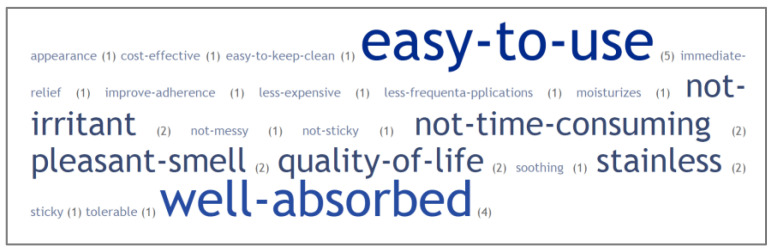
Word cloud representing keywords linked to the patient preference to topical formulations. The difference in character dimension is related to the frequency in Ref. [54].

There is a unanimous consensus that a topical treatment should be personalized in order to meet a patient’s specific needs [53]. As shown in Figure 2, there is a wide variety of subjective factors to lead patient preferences toward a specific topical drug formulations [54]. These factors can also be linked to all five dimensions of adherence classified by the WHO (namely socioeconomic, patients’ related, clinical condition-related, therapy related, and healthcare system-related factors), even if those related to a formulation are among the most recurrent. 

It has been suggested that rheological properties of a topical formulation drive the patient’s preferences and the treatment efficacy, underling the importance of the texture and application characteristics, such as easiness in spreadability and sticky skin feeling. In the case of spreadability, the comparison of the application of four different pharmaceutical vehicles (i.e., a solution, two types of creams at different viscosity, and an ointment) evidenced that volunteers could spread homogeneously only the placebo ointment across the area of the normal skin to be treated [55]. On the other hand, subjects with psoriasis treated for 6 months with different topical formulations (i.e., hydrophobic cream, oleogel, and ointment), revealed higher satisfaction for formulations with a low consistency index, low firmness, and adhesiveness, namely oleogel and hydrophobic creams. Moreover, smell and the effort needed to apply the preparation were less appreciated in ointments [56].

In a clinical study aimed at establishing the patient’s preference among several topical corticosteroids, Felix et al., identifies an extensive variability, confirming the need to individualize the treatment. Subjects expressed the greatest concern for the absorption, messiness, feel on skin, and stains on fabrics; cost and side effects were the two most influential Determinants of Adherence and Quality of Life [57]. More recently, an indirect analysis comparing comments on the use of Cal/BD cream and foam showed that treatment with Cal/BD cream was associated with significantly greater satisfaction on the overall treatment vs. the foam since the cream resulted in ‘ease of application’, ‘not greasy’, and ‘felt moisturizing’ [58]. However, a matching-adjusted indirect comparison approach to compare Cal/BD foam and cream evidences that a Cal/BD foam works more quickly than the cream [59]. Fast response to treatment is important to patients, with rapid improvements in skin symptoms and quality of life, potentially improving treatment adherence, which plays a key role in the effectiveness of topical therapies. 

Hence, the choice on Cal/BD fixed-dose combination products may offer the possibility to tailor the best vehicle for a particular patient on the specific patient’s preference; moreover, physicians and patients should collaborate to determine which option is most likely to improve the skin condition. This would allow the expectation that an improvement in the patient’s adherence would be associated with a lower number of medical consultations, a lower number of patients requiring a systemic therapy, and, therefore, lower total healthcare costs [60].

## 5. Conclusions

Psoriasis is a lifelong disease of underlying autoimmune etiology. Several available treatments can minimize skin lesions and associated symptoms, but cannot provide the complete cure of psoriasis. However, controlling the manifestations of psoriasis significantly improves patients’ quality of life and, above all, the psychological burdens associated with the physical appearance of the psoriatic silvery scales. Topical treatments are first-line, and the fixed-dose combination between Cal and BD is one of the most promising to minimize the risk of skin atrophy due to the use of topical corticosteroids and to speed up the onset of efficacy. Nowadays, different formulations are available on the market, namely ointment, oleoegel, foam, and a cream, to meet the patient’s needs. Among them, the foam is highly accepted, but also the cream seems promising to overcome some limitations related to oleogels and ointments. When hyperkeratosis and acanthosis make the skin an even harder barrier to circumvent, some other strategy can be proposed. In particular, the pioneer work on the use of microneedles and the design of nanocarriers for fixed-drug combination have been opening the path for the development of a series of products for the managing of psoriasis.

## Figures and Tables

**Table 1:** Flux of BD and Cal from commercially available topical formulations determined through in vitro Franz diffusion cells. When the values were not tabulated, the flux is calculated as the slope of the permeation profile curve. NA: not available.

Formulation	Trade Name	Skin Source	Flux (ng/cm^2^ h^−1^)		Reference
			BD	Cal	
Ointment	Daivonex^®^	Pig	NA *	6.7 ± 0.4	[22]
	Diproderm^®^		50 ± 9	NA *	
Ointment	Generic product	Human	2.81 ± 0.28	0.23 ± 0.04	[35]
	Daivobet^®^		1.93 ± 0.29	0.13 ± 0.05	
FoamOintment	Enstillar^®^	Pig	-- *	0.005	Estimated from [27]
	--		-- *	0.002	
Oleogel/suspensionCream	--	Human	~34	~4	[31]
	Wynzora^®^		~68	~12	

* below the limit of quantification.
